# Changes in surgical quality and access after rural hospital closures

**DOI:** 10.1093/haschl/qxaf089

**Published:** 2025-04-25

**Authors:** Cody Lendon Mullens, Patrick L Johnson, Janice C Probst, Justin B Dimick, Andrew M Ibrahim, Adrian Diaz

**Affiliations:** Department of Surgery, University of Michigan, Ann Arbor, MI 48109, United States; Center for Healthcare Outcomes and Policy, Institute for Health Policy and Innovation, Ann Arbor, MI 48109, United States; UM National Clinician Scholars Program, University of Michigan, Ann Arbor, MI 48109, United States; Department of Surgery, University of Michigan, Ann Arbor, MI 48109, United States; Center for Healthcare Outcomes and Policy, Institute for Health Policy and Innovation, Ann Arbor, MI 48109, United States; Arnold School of Public Health, University of South Carolina, Columbia, SC 29208, United States; Department of Surgery, University of Michigan, Ann Arbor, MI 48109, United States; Center for Healthcare Outcomes and Policy, Institute for Health Policy and Innovation, Ann Arbor, MI 48109, United States; Department of Surgery, University of Michigan, Ann Arbor, MI 48109, United States; Center for Healthcare Outcomes and Policy, Institute for Health Policy and Innovation, Ann Arbor, MI 48109, United States; Taubman College of Architecture and Urban Planning, University of Michigan, Ann Arbor, MI 48109, United States; Center for Healthcare Outcomes and Policy, Institute for Health Policy and Innovation, Ann Arbor, MI 48109, United States; Department of Surgery, University of Chicago, Chicago, IL 60637, United States

**Keywords:** rural health, surgical quality, rural hospital closure, hospital closure, access to care

## Abstract

There are rising concerns about the effects of rural hospital closure on access to and quality of care for impacted patients, but little remains known about surgical care. The objective of this study was to evaluate the association of hospital closure with outcomes and access to surgery for common surgical conditions. Using Medicare claims data from 2010–2020, we evaluated the impact of rural hospital closures on surgical quality and access for common operations (colectomy, cholecystectomy, appendectomy, and hernia repair). Using a dynamic difference-in-differences approach, we analyzed 36 884 and 41 185 beneficiaries who lost their nearest and second-nearest rural hospital, respectively. Our findings revealed no significant impacts on surgical quality as measured by 30-day mortality, complications, serious complications, reoperations, and readmissions. While rates of unplanned surgery did not change, median travel distance increased from 13.1 to 16.4 miles for beneficiaries who lost their nearest hospital but was unchanged for those losing their second-nearest hospital. These findings suggest that, while rural hospital closure does not adversely impact surgical quality, it does pose challenges in ensuring access to timely surgical care. Policymakers should consider tailored interventions to mitigate the persistent and growing travel disparities to obtain care in rural America.

## Introduction

Between 2010 and 2024, 151 rural hospitals in the United States have closed.^1^ There are emerging concerns that hospital closures detrimentally impact the availability of emergency and inpatient hospital services.^2-4^ Consequently, there is a growing body of evidence to suggest that rural hospital closures have adversely affected quality of care for common medical conditions.^5,6^ The recent increase in these closures has also led to multiple pieces of federal legislation being both introduced and enacted, which are designed to mitigate existing and prevent future closures through financial support and implementing new models of care.^7,8^

Despite growing concerns around the impacts of rural hospital closures, little is known about the relationship between hospital closure and access and quality of surgical care. Limited evidence evaluating common time-sensitive medical conditions (eg, stroke, myocardial infarction, chronic obstructive pulmonary disease) has suggested higher rates of inpatient mortality in rural health service areas experiencing rural hospital closure.^5^ However, these findings pertained to a single state and were not corroborated in a larger nationally representative sample.^9^ Moreover, less is known about other important inpatient services, such as common surgical conditions, that are frequently treated at rural hospitals.^10-12^ Prior work has demonstrated that barriers in accessing surgery are growing and have strong impacts on quality and cost of surgical care.^13,14^

Our study sought to quantify associations between rural hospital closure and surgical quality and access for common surgical conditions. We evaluated this association using Medicare claims data using a difference-in-differences approach to assess the impact of hospital closure on 30-day postoperative mortality, complications, serious complications, 30-day readmissions, admission type, and changes in travel distance to obtain surgical care. We hypothesized that rural hospital closures would be associated with worse surgical outcomes and longer travel times for patients affected by hospital closure.

## Data and methods

### Data source and population

Data from 100% of claims in the Medicare Provider Analysis and Review (MedPAR) file for calendar years 2010–2020 at nonfederal acute care hospitals were used for this study. Procedure codes for colectomy, cholecystectomy, appendectomy, and incisional hernia repair from the International Classification of Diseases, Ninth and Tenth Revisions (ICD-9 and ICD-10 respectively), Procedure Coding System from the MedPAR file, with confirmatory current procedural terminology codes from the Medicare Carrier File, were used to define the cohort (Appendix Table A1). These specific procedures are commonly performed across the United States and have previously been utilized for evaluating surgical quality among Medicare beneficiaries.^11,15^ Fee-for-service Medicare patients, age 65 years or older, were included in the final analytic cohort. We did not include Medicare Advantage beneficiaries.

Hospital characteristics were obtained from the American Hospital Association (AHA) Annual Survey. MedPAR beneficiary data were linked to AHA data using the unique hospital identification number to specify hospital details where beneficiaries received care.

### Identifying rural hospital closures

To identify rural hospital closures, we used the Cecil G. Sheps Center for Health Services Research Rural Hospital Closures and Conversions tracker.^1^ This tracker is frequently used by researchers to identify rural hospital closures and are identified by collating information from various sources including news alerts, the Centers for Medicare and Medicaid Services (CMS), the Federal Office of Rural Health Policy, the AHA, and the National Rural Health Association. Rural hospitals included in this tracker include those rural hospitals that are any short-term, general acute care, nonfederal hospital located in any nonmetropolitan county, metro census tracts with a Rural-Urban Commuting Area (RUCA) code 4–10, or a large area metro census tract of at least 400 square miles with a population density of 35 or less per square mile and an associated RUCA code of 2–3.

Using unique CMS hospital IDs, we were able to identify and link rural hospital closures to Medicare and AHA data. The Sheps Center tracker distinguishes between rural hospital closures and “conversions.” Hospitals that completely closed no longer provided any health care services, while converted closures no longer provided inpatient care but do continue to provide some long-term/outpatient services (eg, primary care, long-term care, or skilled nursing care).^1^ Because we were evaluating inpatient operations, we included both complete closures and conversions as “closures” for the purpose of this analysis.

### Treatment group

**Figure 1.**
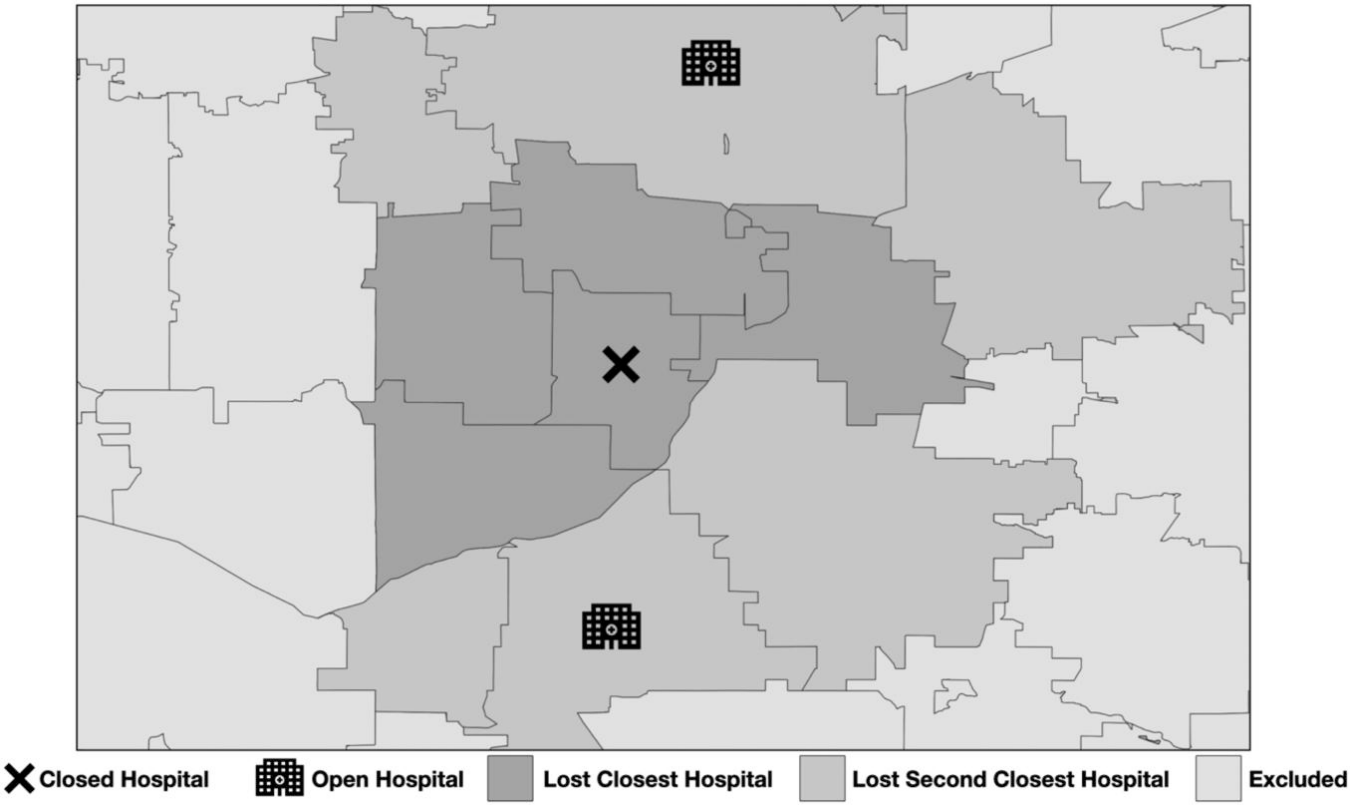
Illustrative example of an empirical approach to assess the impact of losing the nearest or second-nearest hospital due to rural hospital closure. Figure created using ArcGIS.

We used a difference-in-differences approach to evaluate the impacts of rural hospital closures on surgical quality and access. We leveraged a previously validated method to study hospital closures, which evaluates impact based on rural patients losing their nearest vs second-nearest hospital to closure.^6^ Thus, the treatment group for this analysis were beneficiaries whose nearest hospital closed based on their ZIP code of residence. The control group were beneficiaries whose second-nearest hospital closed. To permit geographic analysis, ZIP codes were translated into ZIP Code Tabulation Areas (ZCTAs) using the Health Resources and Services Administration crosswalk. There were 29 beneficiaries (0.04% of the analytic cohort) who had matching or unmatched ZIP codes to ZCTAs who were removed from our analysis. Figure 1 illustrates an example of this empirical approach, which has been validated previously.

### Outcome variables

The primary outcome was 30-day postoperative mortality rate, which was defined by 2 sources as previously described.^16,17^ First, mortality in the hospital was determined by vital status at the time of discharge. Additionally, the Medicare Beneficiary Denominator File was used to ascertain any mortality occurring within 30 days of discharge from the index operation, including patients who died after discharge from their index admission or after transfer to another facility.

Secondary outcomes were complications, serious complications, reoperations, 30-day readmissions, admission type (eg, elective vs unplanned), and changes in travel distance to undergo surgical care. ICD-9 and ICD-10 codes were used to identify postoperative complications such as pulmonary failure, pneumonia, myocardial infarction, deep venous thrombosis, pulmonary embolism, renal failure, surgical site infection, gastrointestinal bleeding, and postoperative hemorrhage (Appendix Table A2). These complications represented a subset of codes from administrative claims with the greatest sensitivity and specificity, as previously described.^15,18,19^ Serious complications were defined as any of the above complications requiring a prolonged length of stay greater than the 75th percentile for the specific procedure performed. This length-of-stay criteria have been applied in multiple previous studies to give clinical face validity (ie, that the complication had a meaningful clinical effect).^15,20-22^ Reoperation was defined as any surgery occurring during the index admission but after the index operation.^23-25^ Readmissions within 30 days of discharge from the index operation were identified using ICD-9 and ICD-10 codes that have previously been used for surgical cohorts.^26^ Admission type was defined as either planned or unplanned surgery. Planned surgery was defined as admissions that were elective. Unplanned surgery was defined as admissions that were urgent or emergent. Travel distances were calculated as the geodetic distance in miles between ZIP code centroids, the most detailed location data available. These distances were calculated for each calendar year to determine changes to travel distances after hospital closure.

### Statistical analysis

We used a dynamic difference-in-differences approach to estimate the association between hospital closure or conversion and surgical outcomes across beneficiaries residing in closure or control ZCTAs, accounting for staggered treatment adoption. Given that treatment timing varied across units, we applied the imputation-based method developed by developed by Borusyak et al,^27^ which addresses potential biases in standard difference-in-differences models arising from heterogeneous treatment effects and staggered treatment initiation.^27^ Each ZCTA's treatment status was determined based on the year it first was exposed to a hospital closure or conversion. For treated ZCTAs, we defined event time as the number of periods relative to the year the exposure was initiated. The ZCTAs where the second-nearest hospital closed were considered untreated units and served as controls, and their event time was not calculated.

We included fixed effects for beneficiary ZCTA, calendar year, gender, race, cohort, and elective admission status to control for time-invariant and unit-specific factors. We also adjusted for patient age and comorbidities using the Elixhauser comorbidity index. The dynamic treatment effects were then estimated for event times ranging from 5 years before to 5 years after hospital closure or conversion.

We tested the parallel trends assumption by examining pretreatment periods to ensure there were no significant differences between treated and control units before treatment initiation. Standard errors were clustered at the ZCTA level to account for within-unit correlation over time. *P* values were from 2-sided tests, with statistical significance deemed as *P* < .05. All analyses were performed using Stata 18 (StataCorp, College Station, TX). After performing this analysis for the overall cohort, we also evaluated specific beneficiary cohorts who underwent planned, elective operations and unplanned (urgent or emergent) operations as sensitivity analyses. This study was deemed exempt by the University of Michigan Institutional Review Board.

### Limitations

This study should be interpreted in the context of its limitations. First, administrative claims data from Medicare lack clinical granularity and do not include younger Americans undergoing surgery. Additionally, this may limit the generalizability of our findings to privately insured rural patients in need of surgical care or those enrolled in Medicare Advantage, which is growing among rural populations.^28^ However, the sample is geographically inclusive, and we studied outcomes that are reliably tracked in Medicare claims data. Further, this older patient population is known to have higher rates of postoperative complications, making this study population more sensitive to identifying changes in quality of surgical care. Second, defining rurality and affected populations by hospital closure is notoriously challenging. To mitigate this, we identified hospital closures using an updated, reliable, and frequently used resource to identify hospital closures and conversions. Additionally, the econometric approach for identifying affected and comparison populations has been validated previously.^6^ Finally, our definition of hospital closures included both rural hospital closures and conversions, which may impact care differently. However, because converted hospitals no longer provided inpatient surgery, they effectively mirror a hospital closure for the purpose of this study.

## Results

### Patient characteristics

Within Medicare beneficiaries undergoing 1 of the 4 studied procedures, we identified 36 884 beneficiaries who lost their nearest and 43 185 who lost their second-nearest rural hospital to closure. Patient characteristics of those who lost their nearest vs second-nearest hospital are summarized in Table 1. The distribution of patient gender was similar (*P* = .78), but a slightly larger proportion of patients who lost their nearest hospital were Black, compared with those losing their second-nearest hospital.

**Table 1.** Patient characteristics of beneficiaries who underwent common operations (appendectomy, cholecystectomy, colectomy, incisional hernia repair) and lost their nearest rural hospital or second-nearest hospital.

Patient characteristics	Lost nearest hospital (*n* = 36 884)	Lost second-nearest hospital (*n* = 43 185)	*P*
Age, median (IQR), y	74 (69–80)	74 (69–80)	.015
Male	16 726 (45.4%)	19 629 (45.5%)	.78
Race			
White	33 536 (91.6%)	39 500 (92.1%)	.004
Black	2370 (6.5%)	2628 (6.1%)	.048
Operative cohort			<.001
Appendectomy	3015 (8.2%)	3742 (8.7%)	
Cholecystectomy	13 321 (36.1%)	15 559 (36.0%)	
Colectomy	14 293 (38.8%)	16 964 (39.3%)	
Incisional hernia	6239 (16.9%)	6907 (16.0%)	
Comorbidities, mean (SD)	3.0 (1.94)	3.01 (1.94)	<.001
Elective admission	16 027 (43.5%)	18 324 (42.4%)	.003
Any complication after surgery	10 217 (27.7%)	11 932 (27.6%)	.82
Serious complications	5057 (13.7%)	5687 (13.2%)	.025
Readmission, 30 days	5451 (14.8%)	6372 (14.8%)	.92
Any reoperation after surgery	3531 (9.6%)	4143 (9.6%)	.93
Mortality, 30 days	2573 (7.0%)	3020 (7.0%)	.93

Data are presented as *n* (%) unless otherwise indicated. Source: Authors’ analysis of 100% of claims from the MedPAR file, 2010–2020.

Abbreviation: MedPAR, Medicare Provider Analysis and Review.

### Hospital characteristics

Of the 56 hospitals included in this study (Appendix Table A3), hospitals had a median of 44 patient beds (95% CI: 25–63.5 beds) and had a low annual operative volume (median: 7; 95% CI: 2.5–20 cases). Approximately half of the hospitals were complete closures, and the other half were converted closures, where inpatient services, including surgery, were eliminated (30 [54%] complete closures and 26 [46%] converted closures). Appendix Table A3 also shows the year of hospital closure for each hospital.

### Changes in surgical quality

**Figure 2.**
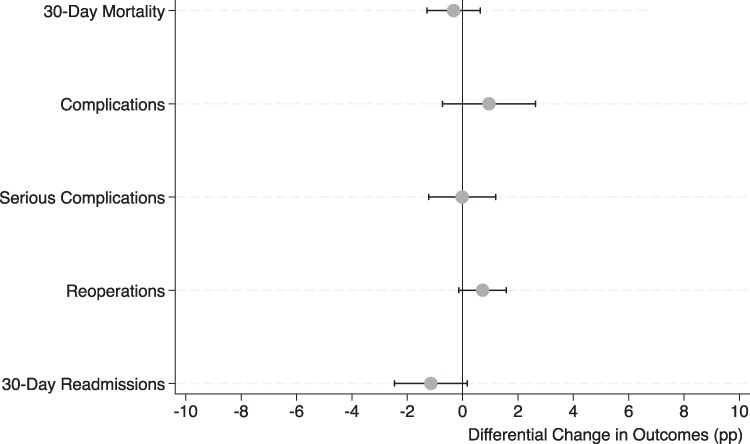
Association between the nearest rural hospital closure and surgical quality and access: 2010–2020. Source: Authors’ analysis of 100% of claims from the MedPAR file: 2010–2020. Abbreviations: MedPAR, Medicare Provider Analysis and Review; pp, percentage points.

Figure 2 demonstrates trend-adjusted difference-in-differences estimates for key measures of surgical quality comparing patients who lost their nearest vs second-nearest rural hospital to closure. Closures of patients’ nearest hospitals were not associated with any adverse quality outcomes compared with patients who lost their second-nearest hospital. For 30-day mortality, the point estimate of change was −0.32% (−1.28% to 0.64%). For complications and serious complications, the change was 0.95% (−0.73% to 2.64%) and 0.02% (−1.23% to 1.20%), respectively. For reoperations, the point estimate of change was 0.72% (−0.14% to 1.58%), and for readmissions the change was 1.14% (−2.46% to 0.17%). These aggregated findings are presented in greater detail in Appendix Figures A1–A6. Sensitivity analyses evaluating beneficiary cohorts who underwent elective surgery and unplanned surgery found results similar to our primary analysis (Appendix Table A4).

### Changes in surgical access

**Figure 3.**
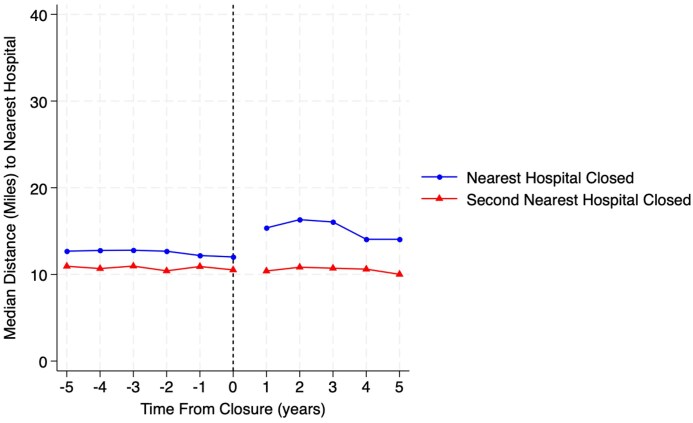
Changes in travel distance to obtain surgical care prior to and after exposure to hospital closure for Medicare beneficiaries who lost their nearest vs second-nearest rural hospital due to closure. Source: Authors’ analysis of 100% of claims from the MedPAR file: 2010–2020. Abbreviation: MedPAR, Medicare Provider Analysis and Review.

Rates of unplanned operations did not significantly increase, with the overall point estimate of change being 0.93% (−1.84% to 3.70%). Changes in travel to obtain surgery did change (Figure 3). Median travel for beneficiaries who lost their nearest hospital increased from 13.1 miles (95% CI: 9.9–17.0) to 16.4 miles (95% CI: 12.1–22.9). For those who lost their second-nearest hospital, the median travel distance remained stable in the pre- and post-closure study period, with distances being 11.5 miles (95% CI: 8.6–15.6) and 11.1 miles (95% CI: 8.5–15.5), respectively.

## Discussion

The present study evaluating surgical quality and access following rural hospital closure has 2 principal findings. First, we found no significant evidence of adverse outcomes (mortality, complications, serious complications, reoperations, or readmissions) among rural patients whose nearest hospital closed. Second, these patients traveled farther to obtain surgical care than their counterparts who did not lose their nearest rural hospital. Taken together, rural hospital closure does not appear to adversely impact patient outcomes from common surgical conditions but has adverse impacts on access to care.

The effects of hospital closure on quality of care for affected patients has been an area of intense research focus without a clear consensus. Using a nationally representative sample for all-comer admissions, Joynt et al^9^ found no evidence of adverse impacts of rural hospital closure on mortality or readmissions at the hospital-service-area level. However, more recent analyses evaluating time-sensitive medical conditions (eg, stroke, myocardial infarction) identified increased mortality among those impacted by rural hospital closure at the hospital-service-area level and nearest-hospital-closure level.^5,6^ Our study extends these analyses in 2 important ways. First, this is the first assessment of the relationships between rural hospital closures and surgical outcomes. Second, we robustly evaluated important measures of quality beyond mortality, including complications, serious complications, readmissions, and reoperations.

An absence of a relationship between rural hospital closure and quality of care may have several underlying explanations. First, the conditions evaluated in this study are common surgical conditions that can be safely performed in multiple settings, including other rural hospitals.^10,11,29^ In addition, although these conditions can be time sensitive, they often have a more generous time window (eg, 24 hours) than their medical counterparts that may need treatment within minutes to hours.^30,31^ Second, there may already be local precedent for residents to travel further for care. Specifically, prior work has suggested that two-thirds of rural patients undergoing low-risk elective surgery bypass a nearby hospital to get their care further away, especially younger patients with private insurance.^32^ Thus, these same communities may already be primed to travel further for surgery when their nearest hospital closes, which may, in part, explain increasing trends in travel time for rural patients to receive surgical care.^33^ Finally, there is evidence in some rural settings that the surgical workforce may begin to erode prior to closing of rural hospitals, which may contribute to patients seeking care elsewhere prior to local rural hospitals shutting their doors.^34^

Travel distance and travel time have also been emphasized when evaluating rural hospital closures. Previous research has linked rural hospital closure to longer travel times and strain of emergency medical services (EMS) transport bandwidth.^2,35-37^ Further, some evidence has suggested that rural hospital closures are associated with lower health care utilization, higher admission rates for ambulatory care–sensitive conditions, poorer access to specialty outpatient services, and overcrowding of nearby hospitals due to spillover effects.^2,38-41^ Our study extends this existing literature to surgical care. Specifically, we found that hospital closure was associated with longer travel time for patients who lost their nearest rural hospital to closure, supporting growing concerns about timeliness and access to surgical care when rural hospitals close.^14^

Our present findings have important implications for policymakers, hospital administrators, and providers. First, although no appreciable harms with respect to surgical quality were uncovered, rural hospital closures likely widen the existing travel disparity these community members are subjected to. Policymakers should consider solutions to address this increased travel burden, including policies to buttress existing EMS transport. Second, for critical-access hospitals that are considering converting to a rural emergency hospital and eliminating inpatient service lines, these findings are timely. Specifically, it appears that patients can have their inpatient surgical care performed elsewhere with similar quality. However, important considerations of converting should be considered, including patient travel, ensuring expedient transfer processes, local relationships, and bandwidth of surrounding hospitals.^42^

Ultimately, rural America is heterogeneous, as are the impacts of rural hospital closure.^2,43^ Thus, a tailored approach rather than one-size-fits-all solutions will be needed to preserve care for rural patients, while balancing both efficiency and equity.^43^ Potential solutions, ranging from travel support for patients to hospital-system reorganization, must incorporate patient-centered approaches to meet community-specific needs when considering how to best support rural populations.

## Conclusion

We identified no appreciable impacts on surgical quality as measured by 30-day mortality, complications, serious complications, reoperations, and readmissions when rural hospitals close. From an access standpoint, while rates of unplanned surgery did not change, median travel distance increased for those whose nearest rural hospital closed. These findings suggest that, while rural hospital closure does not adversely impact surgical quality, it does pose challenges in ensuring access to timely surgical care. Policymakers should consider tailored approaches to mitigating the persistent and growing travel disparities to obtain care in rural America.

## Supplementary Material

qxaf089_Supplementary_Data

## Data Availability

We are unable to share data upon request due to our data use agreement with the Centers for Medicare and Medicaid Services.
