# Supplementary material for: Changes in surgical quality and access after rural hospital closures
Source: Health Aff Sch. 2025 Apr 25;3(5):qxaf089. doi: 10.1093/haschl/qxaf089 (PMC12048781; doi:10.1093/haschl/qxaf089)
Supplement: qxaf089_Supplementary_Data [file qxaf089_supplementary_data.zip › Appendix exhibits R&R1.docx]

**Appendix Table 1:** ICD-9 and -10 coding strategy for common general surgery procedures evaluated in this study.

| **Appendectomy** | | **Cholecystectomy** | | **Colectomy** | | | **Hernia repair** | |
| --- | --- | --- | --- | --- | --- | --- | --- | --- |
| *ICD-9* | *ICD-10* | *ICD-9* | *ICD-10* | *ICD-9* | *ICD-10* | | *ICD-9* | *ICD-10* |
| 470 | 0DTJ0ZZ | 512 | 0F540ZZ | 1731 | 0DBE0ZZ | 0DBN0ZZ | 5351 | 0WQF0ZZ |
| 4701 | 0DTJ4ZZ | 5121 | 0F543ZZ | 1732 | 0DBE3ZZ | 0DBN3ZZ | 5359 | 0WQF3ZZ |
| 4709 | 0DTJ7ZZ | 5122 | 0FB40ZZ | 1733 | 0DBE4ZZ | 0DBN4ZZ | 5361 | 0WQF4ZZ |
| 4711 |  | 5123 | 0FB43ZZ | 1734 | 0DBE7ZZ | 0DBN7ZZ | 5362 | 0WUF07Z |
| 4719 |  | 5124 | 0FT40ZZ | 1735 | 0DBF0ZZ | 0DTE0ZZ | 5363 | 0WUF0JZ |
|  |  |  | 0FT44ZZ | 1736 | 0DBF3ZZ | 0DTE4ZZ | 5369 | 0WUF0KZ |
|  |  |  | 0F544ZZ | 1737 | 0DBF4ZZ | 0DTE7ZZ |  | 0WUF47Z |
|  |  |  | 0FB44ZZ | 1738 | 0DBF7ZZ | 0DTF0ZZ |  | 0WUF4JZ |
|  |  |  |  | 1739 | 0DBG0ZZ | 0DTF4ZZ |  | 0WUF4KZ |
|  |  |  |  | 1741 | 0DBG3ZZ | 0DTF7ZZ |  |  |
|  |  |  |  | 1742 | 0DBG4ZZ | 0DTG0ZZ |  |  |
|  |  |  |  | 4570 | 0DBG7ZZ | 0DTG4ZZ |  |  |
|  |  |  |  | 4571 | 0DBH0ZZ | 0DTG7ZZ |  |  |
|  |  |  |  | 4572 | 0DBH3ZZ | 0DTH0ZZ |  |  |
|  |  |  |  | 4573 | 0DBH4ZZ | 0DTH4ZZ |  |  |
|  |  |  |  | 4574 | 0DBH7ZZ | 0DTH7ZZ |  |  |
|  |  |  |  | 4575 | 0DBK0ZZ | 0DTK0ZZ |  |  |
|  |  |  |  | 4576 | 0DBK3ZZ | 0DTK4ZZ |  |  |
|  |  |  |  | 4579 | 0DBK4ZZ | 0DTK7ZZ |  |  |
|  |  |  |  | 4580 | 0DBK7ZZ | 0DTL0ZZ |  |  |
|  |  |  |  | 4581 | 0DBL0ZZ | 0DTL4ZZ |  |  |
|  |  |  |  | 4582 | 0DBL3ZZ | 0DTL7ZZ |  |  |
|  |  |  |  | 4583 | 0DBL4ZZ | 0DTM0ZZ |  |  |
|  |  |  |  |  | 0DBL7ZZ | 0DTM4ZZ |  |  |
|  |  |  |  |  | 0DBM0ZZ | 0DTM7ZZ |  |  |
|  |  |  |  |  | 0DBM3ZZ | 0DTN0ZZ |  |  |
|  |  |  |  |  | 0DBM4ZZ | 0DTN4ZZ |  |  |
|  |  |  |  |  | 0DBM7ZZ | 0DTN7ZZ |  |  |

**Appendix Table A2:** List of Post Operative complications and ICD codes

| Pulmonary failure | 9604 9605 967 9670 51881 51882 51884 T364X1A T361X1A T423X1A J9600 J80 J9620 J9601 R0603 J9621 J9602 J9622 J9690 J9691 J9692 |
| --- | --- |
| Pneumonia | 481 482 483 484 485 5070 J13 J181 J150 J151 J14 J154 J153 J154 J1520 J15211 J15212 J1529 J158 J155 J156 A481 J158 J159 J157 J160 J168 B250 A3701 A3711 A3781 A3791 A221 B440 J17 B7781 J17 J180 J690 J691 J698 |
| Myocardial Infarction | 410 I2109 I220 I2101 I2102 I2109 I220 I2119 I221 I2111 I221 I2119 I221 I2129 I228 I2129 I228 I214 I222 I2121 I2129 I228 I219 I21A1 I21A9 I213 I219 I21A1 I21A9 I229 I219 I21A1 I21A9 |
| DVT/PE | 4151 4512 4538 45111 45119 45181 387 I2601 I2690 I2602 I2692 I2609 I2699 I803 I82611 I82612 I82613 I82619 I82621 I82622 I82623 I82629 I82601 I82602 I82603 I82609 I82A11 I82A12 I82A13 I82A19 I82B11 I82B12 I82B13 I82B19 I82C11 I82C12 I82C13 I82C19 I82210 I82290 I82890 I8290 I8010 I8011 I8012 I8013 I80201 I80202 I80203 I80209 I80221 I80222 I80223 I80229 I80231 I80232 I80233 I80239 I80291 I80292 I80293 I80299 I80211 I80212 I80213 I80219 |
| Acute Renal Failure | 584 3995 N170 N171 N172 N178 N179 Z992 |
| Hemorrhage | 9981 D7801 D7802D7821 D7822 E3601 E3602 E89810 E89811 G9731 G9732 G9751 G9752 H59111 H59112 H59113 H59119 H59121 H59122 H59123 H59129 H59311 H59312 H59313 H59319 H59321 H59322 H59323 H59329 H9521 H9522 H9541 H9542 I97410 I97411 I97418 I9742 I97610 I97611 I97618 I97620 J9561 J9562 J95830 J95831 K9161 K9162 K91840 K91841 L7601 L7602 L7621 L7622 M96810 M96811 M96830 M96831 N9961 N9962 N99820 N99821 D7801 D7802 D7831 D7832 E3601 E3602 E89820 H59331 D7833 E89821 G9731 G9732 G9761 G9762 H59111 H59112 H59113 H59119 H59121 H59122 H59123 H59129 H59332 H59333 H59339 H59341 H59342 H59343 H59349 H9521 H9522 H9551 H9552 I97410 I97411 I97418 I9742 I97621 I97630 I97631 I97638 J9561 J9562 J95860 J95861 K9161 K9162 K91870 K91871 L7601 L7602 L7631 L7632 M96810 M96811 M96840 M96841 N9961 N9962 N99840 N99841 D7834 E89822 E89823 G9763 G9764 H59351 H59352 H59353 H59359 H59361 H59362 H59363 H59369 H9553 H9554 I97622 I97640 I97641 I97648 J95862 J95863 K91872 K91873 L7633 L7634 M96842 M96843 N99842 N99843 |
| Surgical Site Infection | 9583 9983 9985 5491 T798XXA T8130XA T8132XA T8131XA T8133XA K6811 T814XXA |
| GI Hemorrhage | 5310 5311 5312 5314 5316 5320 5321 5322 5324 5326 5330 5331 5332 5334 5336 5340 5341 5342 5344 5346 53082 53501 53511 53521 53531 53541 53551 53561 5789 K250 K251 K252 K254 K256 K260 K261 K262 K264 K266 K270 K271 K272 K274 K276 K280 K281 K282 K284 K286 K228 K2901 K2931 K2941 K2951 K2961 K2921 K2931 K2961 K2971 K2991 K2981 K922 |

**Appendix Table A3**: Characteristics of rural hospitals that closed during the study period. Data source: Cecil G. Sheps Center for Health Services Research Rural Hospital Closures and Conversions trackers and American Hospital Association Annual Survey data; 2010-2020.

| **Rural Hospital Closures** | **Total** |
| --- | --- |
|  | **N=56** |
| Annual Operative Volume, Median (IQR) | 7 (2.5-20) |
| # of Beds, Median (IQR) | 44 (25-63.5) |
| *Type of Closure* |  |
| Complete Closure | 30 (54%) |
| Converted Closure | 26 (46%) |
| *Closure Year* |  |
| 2011 | 4 (7%) |
| 2012 | 5 (9%) |
| 2013 | 5 (9%) |
| 2014 | 4 (7%) |
| 2015 | 13 (23%) |
| 2016 | 5 (9%) |
| 2017 | 5 (9%) |
| 2018 | 5 (9%) |
| 2019 | 10 (18%) |

Note: IQR – interquartile range.

**Appendix Figure A1:** Changes in 30-day mortality rates from common general surgery operations among Medicare beneficiaries between 2010-2020 based on loss of nearest rural hospital as exposure.


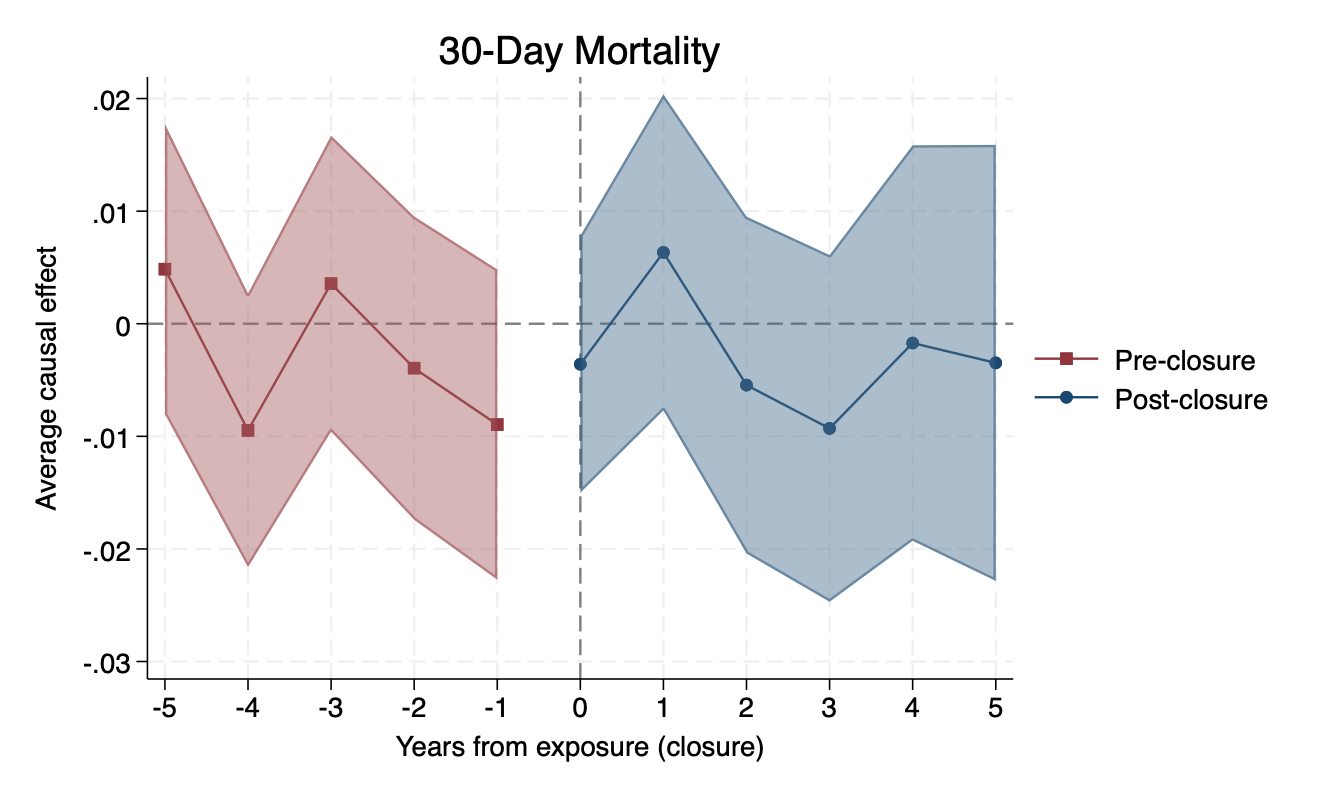


**Appendix Figure A2:** Changes in complication rates from common general surgery operations among Medicare beneficiaries between 2010-2020 based on loss of nearest rural hospital as exposure.


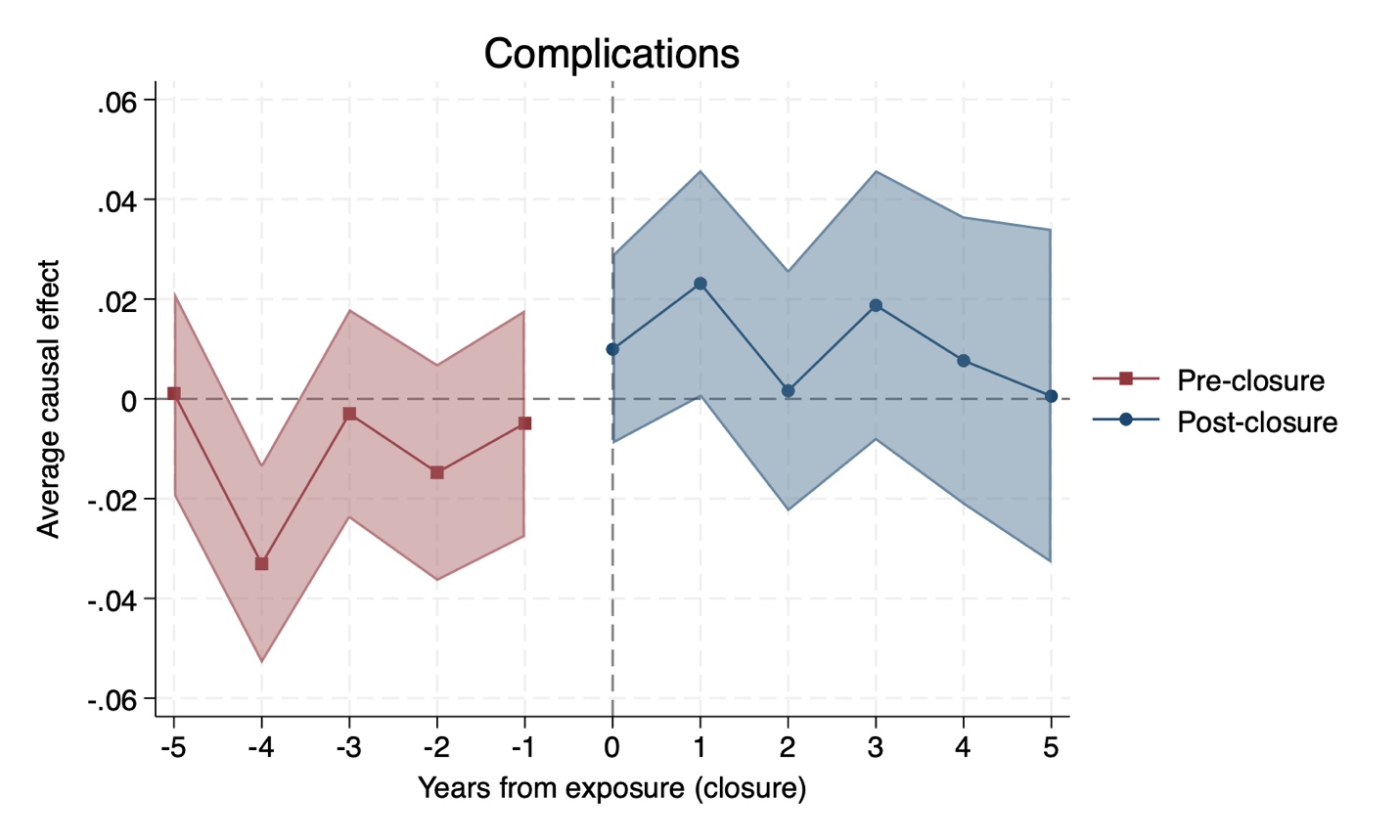


**Appendix Figure A3:** Changes serious complication rates from common general surgery operations among Medicare beneficiaries between 2010-2020 based on loss of nearest rural hospital as exposure.


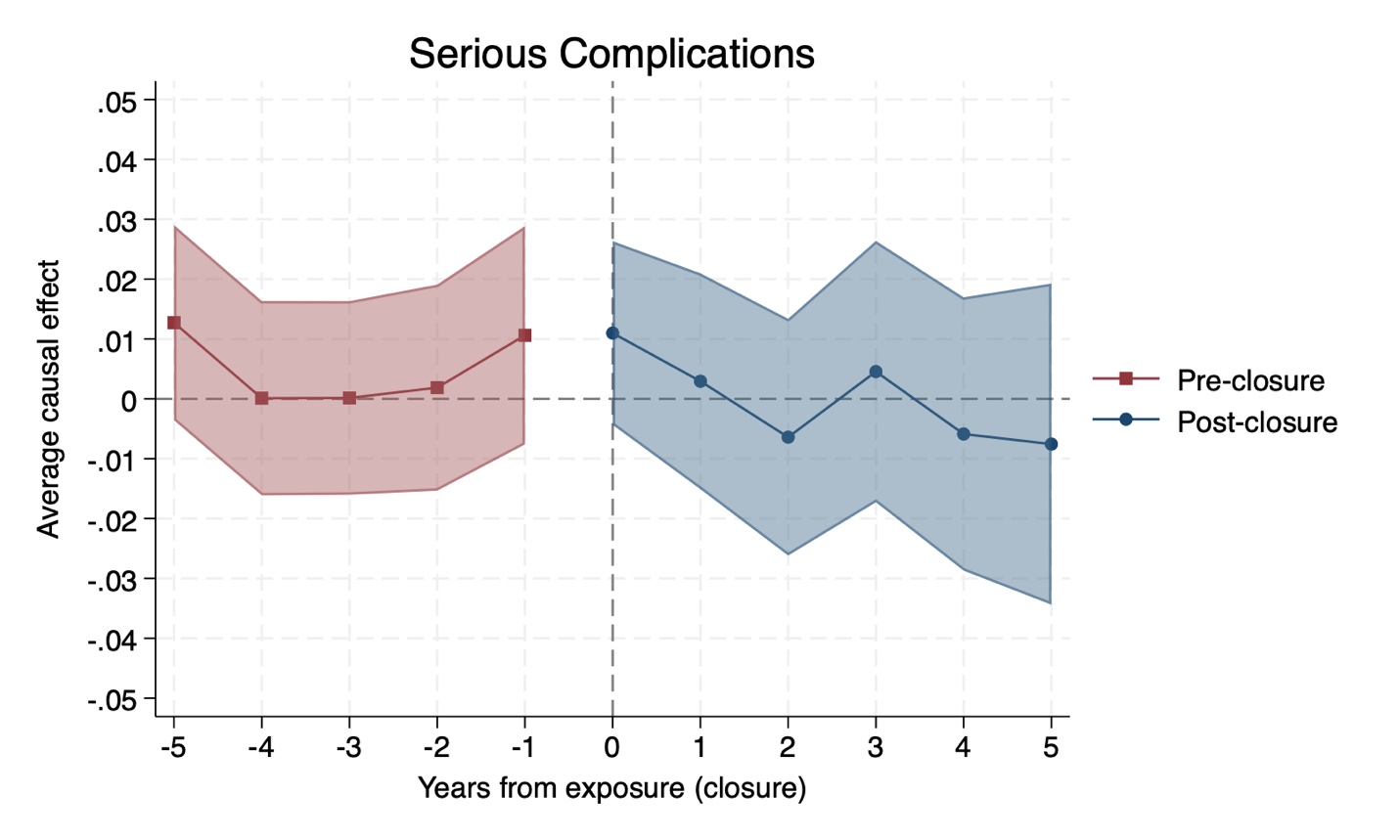


**Appendix Figure A4:** Changes reoperation rates rates for common general surgery operations among Medicare beneficiaries between 2010-2020 based on loss of nearest rural hospital as exposure.


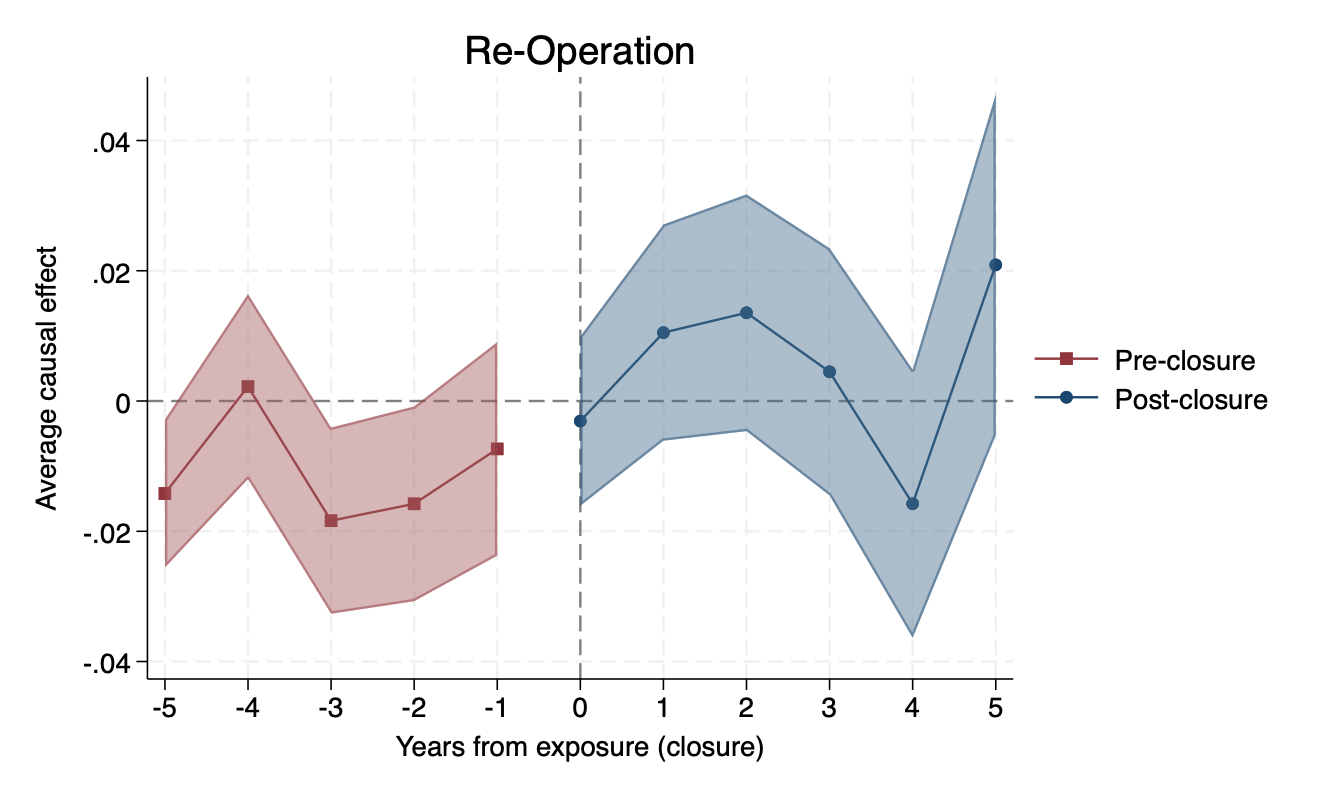


**Appendix FigureA5:** Changes in 30-day readmission rates from common general surgery operations among Medicare beneficiaries between 2010-2020 based on loss of nearest rural hospital as exposure.


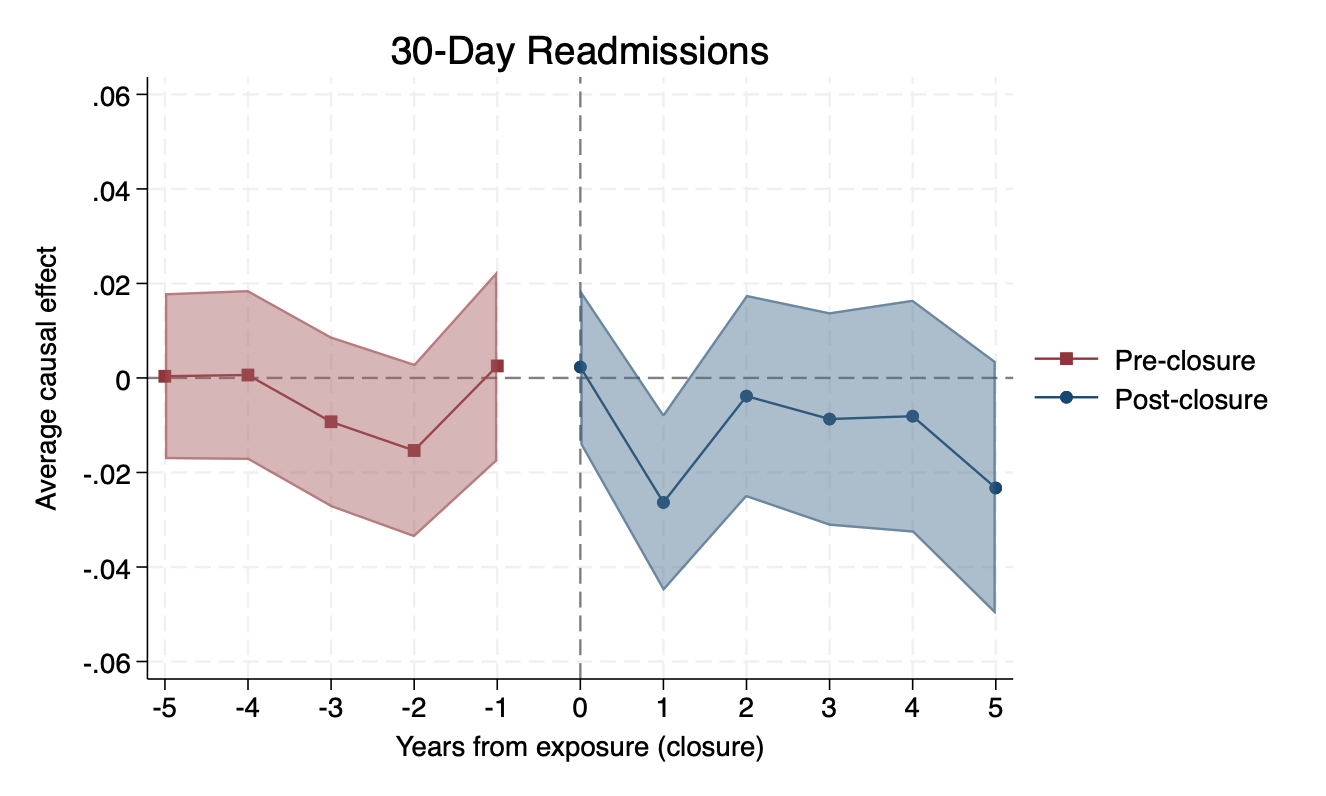


**Appendix Figure A6:** Changes in elective admissions for common general surgery operations among Medicare beneficiaries between 2010-2020 based on loss of nearest rural hospital as exposure.


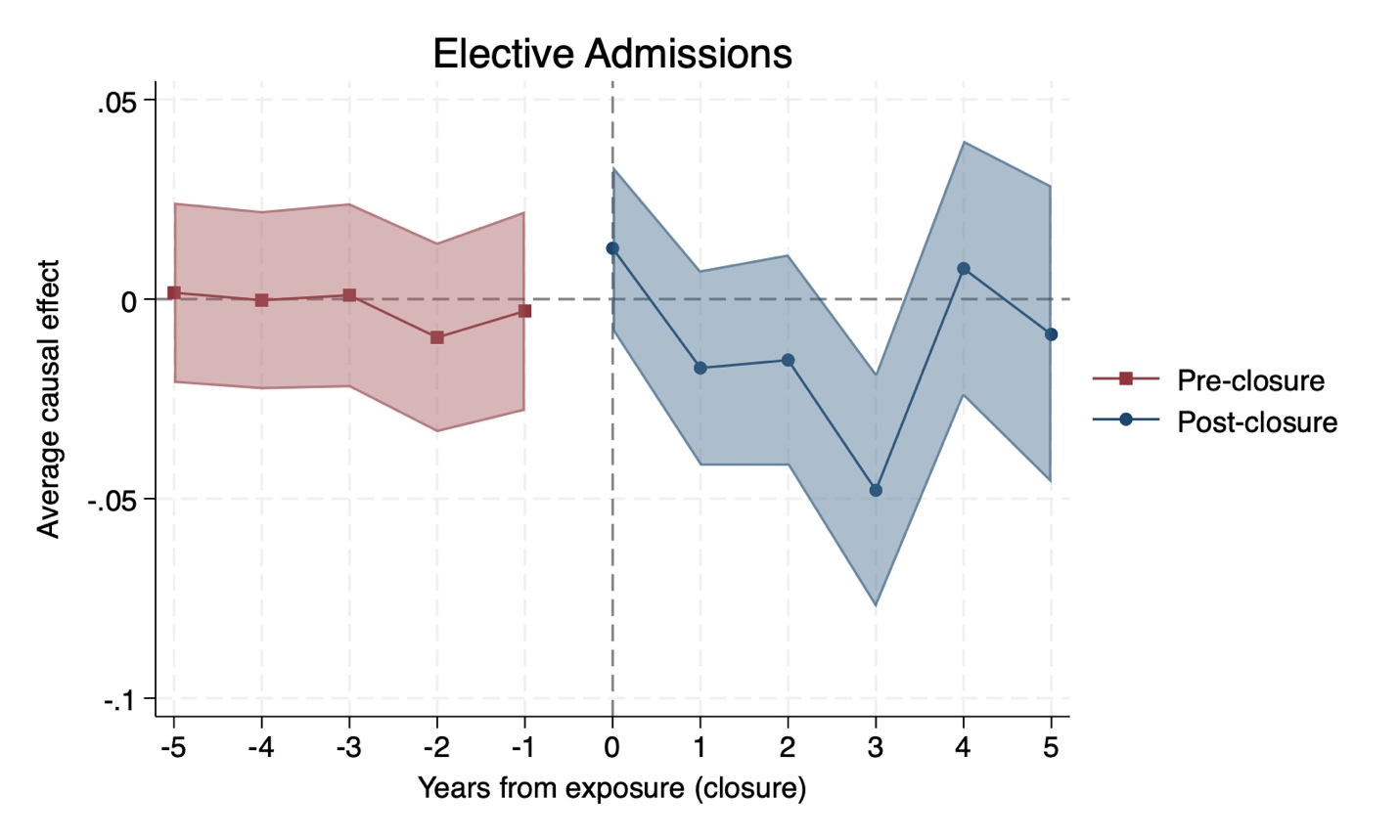


**Appendix Table A4**: Sensitivity analysis for estimated treatment effects among beneficiaries who underwent elective procedures and unplanned procedures.

| **Elective** | | | | |
| --- | --- | --- | --- | --- |
| **Outcome** | **Estimated treatment effect** | **Lower bound** | **Upper bound** | **P** |
| 30-Day Mortality | -0.0097012 | -0.020377 | 0.0009747 | 0.075 |
| Complications | 0.0017034 | -0.0184194 | 0.0218262 | 0.868 |
| Serious Complications | -0.0058318 | -0.0188717 | 0.0072081 | 0.381 |
| Reoperations | 0.0081638 | -0.0056184 | 0.021946 | 0.246 |
| 30-Day Readmissions | -0.0143799 | -0.0331007 | 0.0043409 | 0.132 |
| **Unplanned (Urgent and Emergent)** | | | | |
| 30-Day Mortality | -0.0001457 | -0.0148656 | 0.0145743 | 0.985 |
| Complications | 0.0067748 | -0.0160616 | 0.0296112 | 0.561 |
| Serious Complications | -0.0036674 | -0.0237009 | 0.0163661 | 0.72 |
| Reoperations | 0.005392 | -0.0057453 | 0.0165293 | 0.343 |
| 30-Day Readmissions | -0.012299 | -0.0302777 | 0.0056798 | 0.18 |
